# Effect of driving pressure on mortality in ARDS patients during lung protective mechanical ventilation in two randomized controlled trials

**DOI:** 10.1186/s13054-016-1556-2

**Published:** 2016-11-29

**Authors:** Claude Guérin, Laurent Papazian, Jean Reignier, Louis Ayzac, Anderson Loundou, Jean-Marie Forel, on behalf of the investigators of the Acurasys and Proseva trials

**Affiliations:** 1 Réanimation Médicale Groupement Hospitalier Nord Hospices civils de Lyon, Lyon, France; 2 Université de Lyon, 1 69100, Villeurbanne, France; 3 Institut Mondor de Recherche Biomédicale, INSERM 955 Equipe 13, Créteil, France; 4 Hôpitaux de Marseille Hôpital Nord Réanimation des Détresses Respiratoires et des Infections Sévères, Marseille, 13015 France; 5 Aix-Marseille University EA 3279 Research Unit Department of Public Health Medecine School University Marseille, Marseille, France; 6 URMITE UMR CNRS 7278, Marseille, 13005 France; 7 Réanimation Médicale CHU de Nantes, Nantes, France; 8 Centre de Coordination et de Lutte Contre les Infections Nosocomiales Sud-Est, Saint-Genis Laval, France; 9 Hôpitaux de Marseille Department of Research and Innovation Support Unit for clinical research and economic evaluation, Marseille, 13005 France

**Keywords:** ARDS, Prone position, Neuromuscular blocking agents, Driving pressure, Compliance, Plateau pressure, Lung protective ventilation

## Abstract

**Background:**

Driving pressure (ΔPrs) across the respiratory system is suggested as the strongest predictor of hospital mortality in patients with acute respiratory distress syndrome (ARDS). We wonder whether this result is related to the range of tidal volume (V_T_). Therefore, we investigated ΔPrs in two trials in which strict lung-protective mechanical ventilation was applied in ARDS. Our working hypothesis was that ΔPrs is a risk factor for mortality just like compliance (Crs) or plateau pressure (Pplat,rs) of the respiratory system.

**Methods:**

We performed secondary analysis of data from 787 ARDS patients enrolled in two independent randomized controlled trials evaluating distinct adjunctive techniques while they were ventilated as in the low V_T_ arm of the ARDSnet trial. For this study, we used V_T_, positive end-expiratory pressure (PEEP), Pplat,rs, Crs, ΔPrs, and respiratory rate recorded 24 hours after randomization, and compared them between survivors and nonsurvivors at day 90. Patients were followed for 90 days after inclusion. Cox proportional hazard modeling was used for mortality at day 90. If colinearity between ΔPrs, Crs, and Pplat,rs was verified, specific Cox models were used for each of them.

**Results:**

Both trials enrolled 805 patients of whom 787 had day-1 data available, and 533 of these survived. In the univariate analysis, ΔPrs averaged 13.7 ± 3.7 and 12.8 ± 3.7 cmH_2_O (*P* = 0.002) in nonsurvivors and survivors, respectively. Colinearity between ΔPrs, Crs and Pplat,rs, which was expected as these variables are mathematically coupled, was statistically significant. Hazard ratios from the Cox models for day-90 mortality were 1.05 (1.02–1.08) (*P* = 0.005), 1.05 (1.01–1.08) (*P* = 0.008) and 0.985 (0.972–0.985) (*P* = 0.029) for ΔPrs, Pplat,rs and Crs, respectively. PEEP and V_T_ were not associated with death in any model.

**Conclusions:**

When ventilating patients with low V_T_, ΔPrs is a risk factor for death in ARDS patients, as is Pplat,rs or Crs. As our data originated from trials from which most ARDS patients were excluded due to strict inclusion and exclusion criteria, these findings must be validated in independent observational studies in patients ventilated with a lung protective strategy.

**Trial registration:**

Clinicaltrials.gov NCT00299650. Registered 6 March 2006 for the Acurasys trial.

Clinicaltrials.gov NCT00527813. Registered 10 September 2007 for the Proseva trial.

**Electronic supplementary material:**

The online version of this article (doi:10.1186/s13054-016-1556-2) contains supplementary material, which is available to authorized users.

## Background

Lung protective ventilation, which is a current strong recommendation in patients with the acute respiratory distress syndrome (ARDS), includes several components, the most important of them being lowering tidal volume (V_T_) and limiting plateau (Pplat,rs) equal to or below 30 cm H_2_O. This combined strategy is indeed the single ventilator intervention that has been shown to significantly improve survival so far [1]. This trial introduced scaling of V_T_ to the predicted body weight. However, in the lower V_T_ group with the significant improvement in survival, V_T_ was not strictly set to 6 ml/kg but may have been accommodated in the range 4–8 ml/kg. V_T_ is equal to the difference between plateau (Pplat,rs) and total positive end-expiratory pressure (PEEPtot,rs) measured at zero flow divided by the compliance of the respiratory system (Crs). The difference between Pplat,rs and PEEPtot,rs is the driving pressure (ΔPrs). Therefore, using ΔPrs to select V_T_ is equivalent to titrating V_T_ for Crs, as V_T_ is equal to ΔPrs divided by Crs.

The first report suggesting that ΔPrs is associated with mortality came from the study by Amato et al. [2]. Recently a retrospective analysis of several trials in patients with ARDS comparing different PEEP levels at the same V_T_ or different V_T_ levels at the same PEEP, or a combination of both, found that ΔPrs is the stronger predictor of mortality as compared with Pplat,rs [3]. Furthermore, the relative risk of mortality significantly increased above a threshold in the vicinity of 15 cm H_2_O. It is important to emphasize at this point that the threshold of a driving pressure of 14 or 15 cmH_2_O to predict outcome or titrate V_T_ has not been validated or confirmed. With the aim of attempting to confirm this finding, we also wondered whether this result may be due to the wide range of V_T_ used across the trials included. To try to answer this question, we investigated ΔPrs from two trials [4, 5] in which strict lung-protective mechanical ventilation, in particular 6 ml/kg predicted body weight V_T_, was applied to patients with ARDS. Our working hypothesis was that ΔPrs was associated with death, just like compliance (Crs) or Pplat,rs.

## Methods

This is a secondary analysis of patients enrolled in two previously published randomized controlled trials, namely Acurasys [4] and Proseva [5]. The first trial compared the neuromuscular blocking agent (NMBA) cisatracurium to placebo and the second compared the prone to the supine position. Both had similar inclusion criteria (notably early ARDS and partial pressure of oxygen in arterial blood (PaO_2_)/oxygen fraction in air (F_I_O_2_) <150 mm Hg under PEEP ≥5 cm H_2_O) and similar lung-protective mechanical ventilation (in particular 6 ml/kg predicted body weight V_T_, limited Pplat,rs and PEEP/F_I_O_2_ table [1]). ARDS was identified based on the American-European consensus definition criteria [6]. Both trials identified significant survival benefit in the experimental group.

From the case report form of each original trial, we extracted the relevant variables for the present study, namely sequential organ failure assessment (SOFA) score, continuous NMBA infusion, prone position, pH, partial pressure of carbon dioxide in arterial blood (PaCO_2_), PaO_2_/F_I_O_2_, lactate, respiratory rate, V_T_, PEEP, Pplat,rs, Crs and ΔPrs, which were recorded at day 1 as the values corresponding to those gathered 24 hours after randomization in each trial. In addition, we used gender, age and simplified acute physiology score (SAPS) II recorded at the time of admission and compared between survivors and nonsurvivors at day 90. The allocation assignment in the two trials was entered as predefined covariates into the models. Furthermore, as recent experimental data suggest that the amount of energy transferred from the ventilator into the lung may be a contributing factor to ventilator-induced lung injury (VILI) [7], we computed the mechanical power as ΔPrs × V_T_ × respiratory rate. It was expressed as J/min and was included in the analysis in the present study.

Descriptive statistics included percentages for categorical variables and means and standard deviation (SD) for continuous variables and were compared using nonparametric tests. Cox proportional hazard models were used with covariates significantly different between survivors and non survivors at the threshold of 0.20 and mortality at day 90 as the dependent variable. Even though ΔPrs, Crs, and Pplat,rs are mathematically coupled, we planned to formally test the collinearity within them and, if verified, to use a specific Cox model for each. Because pH and lactate interact, we used their interaction term in the Cox models. We also included those collinear variables two-by-two into four additional Cox regression models, besides the other covariates. One model pertained to Pplat,rs and ΔPrs, one to Crs and ΔPrs, one to ΔPrs and mechanical power, and one to Crs and Pplat,rs. Two interpretations of the results could *a priori* be deciphered. If both variables in the couple lacked significance, the conclusion could be that the same information was carried by each component of the couple. If one of the variables in the couple remained significantly correlated with survival, this variable would be more informative than the other in the couple. Univariate and multivariate Cox proportional hazard regression models were used to estimate the hazard ratio (HR).

Kaplan-Meier graphs were used to express the probability of death from inclusion to day 90 and were compared across groups by the log rank test. Groups were defined from the median values in the present cohort. We split ΔPrs into five quintiles of almost 150 patients each following the method used in both the Amato [3] and the Lung Safe [8] studies by using the Ntiles function in SPSS software. Comparison between quintiles was made by analysis of variance (ANOVA) with post-hoc comparison from the first quintile performed using the Tukey test. A *p* value <0.05 was considered significant. The statistical analysis was conducted using IBM SPSS Statistics, version 20.0 (IBM SPSS Inc., Chicago, IL, USA).

## Results

A total of 805 patients were included in the two trials, of these patient, 787 had data available at day 1. There were 533 survivors and 254 non-survivors at day 90 (mortality rate 32.3% for the combined trials). The comparison between survivors and non survivors at day 90 is shown in Table 1.

**Table 1:** Characteristics at the time of inclusion or day 1 between survivors and non-survivors at day 90

Variables	All (*n* = 787)	Survivors (*n* = 533)	Nonsurvivors (*n* = 254)	*P*
Male gender	542 (68.9)	366 (68.7)	176 (69.3)	0.923
Age, years	59 ± 16	56 ± 15	66 ± 14	<0.001
SAPS II on ICU admission	45 ± 15	45 ± 16	51 ± 15	<0.001
SOFA score on day 1	7 ± 4	7 ± 4	9 ± 4	<0.001
Continuous NMBA as allocation group	173 (22.0)	117 (22.0)	56 (22.0)	0.976
Prone position as allocation group	233 (34.4)	181 (34.0)	52 (20.5)	<0.001
Arterial pH on day 1	7.35 ± 0.09	7.36 ± 0.08	7.32 ± 0.10	<0.001
PaCO_2_ on day 1, mmHg	47 ± 11	46 ± 11	47 ± 11	0.076
PaO_2_/FIO_2_ ratio on day 1	159 ± 74	163 ± 76	152 ± 68	0.056
Lactate on day 1, mmol/L	2.0 ± 1.9	1.8 ± 1.6	2.4 ± 2.2	<0.001
Respiratory rate on day 1,/minute	27 ± 6	26 ± 5	27 ± 6	0.010
Tidal volume on day 1, ml	397 ± 76	398 ± 76	395 ± 78	0.413
Tidal volume on day 1, ml/PBW kg	6.3 ± 0.8	6.2 ± 0.8	6.3 ± 0.8	0.691
PEEP on day 1, cm H2O	10 ± 3	10 ± 3	10 ± 3	0.210
Plateau pressure on day 1, cm H_2_O	23 ± 4	23 ± 4	24 ± 4	<0.001
Tidal compliance on day 1, ml/cm H_2_O	33 ± 12	34 ± 12	31 ± 12	0.016
Driving pressure on day 1, cm H_2_O	13 ± 4	13 ± 4	14 ± 4	0.002
Mechanical power on day 1, J/min	13.4 ± 5.0	13.0 ± 4.8	14.3 ± 5.4	<0.001

Quantitative values are expressed as mean ± SD and qualitative values are numbers (percentage of group). Tidal compliance of respiratory system was calculated as the ratio of tidal volume to driving pressure. Driving pressure was calculated as the difference between plateau pressure and applied positive end-expiratory pressure (PEEP). Mechanical power was calculated as the product of driving pressure in Newton (cm H2O × 0.098), tidal volume and respiratory rate. Day 1 was defined as the 24 hours following the inclusion. *ICU* intensive care unit, *SAPS II* simplified acute physiology score II, *SOFA* sequential organ failure assessment, *PaO*_*2*_ */FIO*_*2*_ *ratio* the ratio of the partial pressure of arterial oxygen to the fraction of inspired oxygen, *PaCO*_*2*_ partial pressure of arterial carbon dioxide, *PBW* predicted body weight, *NMBA* neuromuscular blocking agent

As the collinearity between ΔPrs, mechanical power, Pplat,rs and Crs was statistically significant, a Cox model was constructed for each of these variables. The Cox model pertaining to ΔPrs is shown in Table 2. Age, SOFA, prone position, pH, lactate, pH and its interaction with lactate and ΔPrs were significantly associated with the outcome at day 90 whilst NMBA was not. For each of the additional three Cox models that included mechanical power, Pplat,rs, or Crs as a single covariable, the significant predictors of patient outcome were the same as for ΔPrs (see additional files 1, 2 and 3). The HR was high for lactate in each Cox model, with wide confidence intervals (Table 2 and Additional files 1, 2 and 3). After multiple adjustments of coupled variables, four additional Cox models were performed (Additional file 4). ΔPrs and Pplat,rs remained significantly associated with patient outcome, meaning that each of them brought specific and distinct information (model 1 in Additional file 4). For ΔPrs and mechanical power, ΔPrs maintained a significant association with mortality at day 90, and hence carries specific information (model 2 in Additional file 4). However, for ΔPrs and Crs, and for Pplat,rs and Crs (models 3 and 4, respectively, in Additional file 4), neither of the variables in each pair were statistically significant. Therefore, it could be concluded that ΔPrs and Crs, on one hand, and Pplat,rs and Crs on the other hand, share the same information.

**Table 2:** Multivariate Cox regression analysis for factors including driving pressure at day 1 associated with ARDS mortality at day 90

Variables	Hazard ratio (95% CI)	*p*
Age, per year	1.04 (1.03–1.05)	<0.001
SOFA score on day 1, per unit	1.07 (1.03–1.11)	<0.001
Continuous NMBA as allocation group, (reference is yes)	0.64 (0.45–0.91)	0.012
Continuous prone position as allocation group (reference is yes)	0.68 (0.47–0.98)	0.037
Respiratory rate on day 1, per unit	1.01 (0.98–1.03)	0.698
PaO_2_/FiO_2_ on day 1, per unit	1.00 (0.99–1.01)	0.831
Arterial pH on day 1, per unit	0.057 (0.009–0.371)	0.003
Lactate on day 1, per unit	18.44 (1.39–244.00)	0.027
Interaction between lactate and arterial pH on day 1, per unit	0.67 (0.47–0.96)	0.030
Driving pressure on day 1, per unit	1.05 (1.02–1.08)	0.005

Driving pressure was calculated as the difference between plateau pressure and applied positive end-expiratory pressure (PEEP). Day 1 was defined as the 24 hours following the inclusion. *CI* confidence intervals, *SOFA* sequential organ failure assessment, *NMBA* neuromuscular blocking agent, *PaO*_*2*_ */FIO*_*2*_ ratio of the partial pressure of arterial oxygen to the fraction of inspired oxygen

**Fig. 1:**
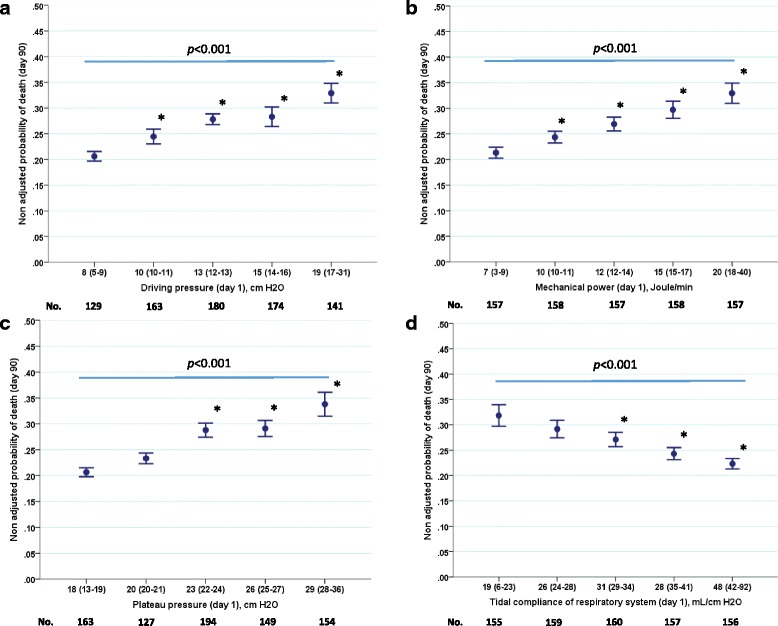
Unadjusted mortality at day 90 across quintiles of driving pressure (**a**), mechanical power (**bb**), Pplat,rs (**c**) and Crs (**d**). The *bars* are standard error of the mean (SEM). The numbers below the *x axis* are the numbers of patients in each quintile. *P* < 0.001 across quintiles (analysis of variance). *P < 0.05 versus the first quintile

Figure 1 displays the unadjusted mortality rates at day 90 across five quintiles of ΔPrs (Fig. 1a), mechanical power (Fig. 1b), Pplat,rs (Fig. 1c) and Crs (Fig. 1d). No distinct threshold of ΔPrs was identified (Fig. 1).

**Fig. 2:**
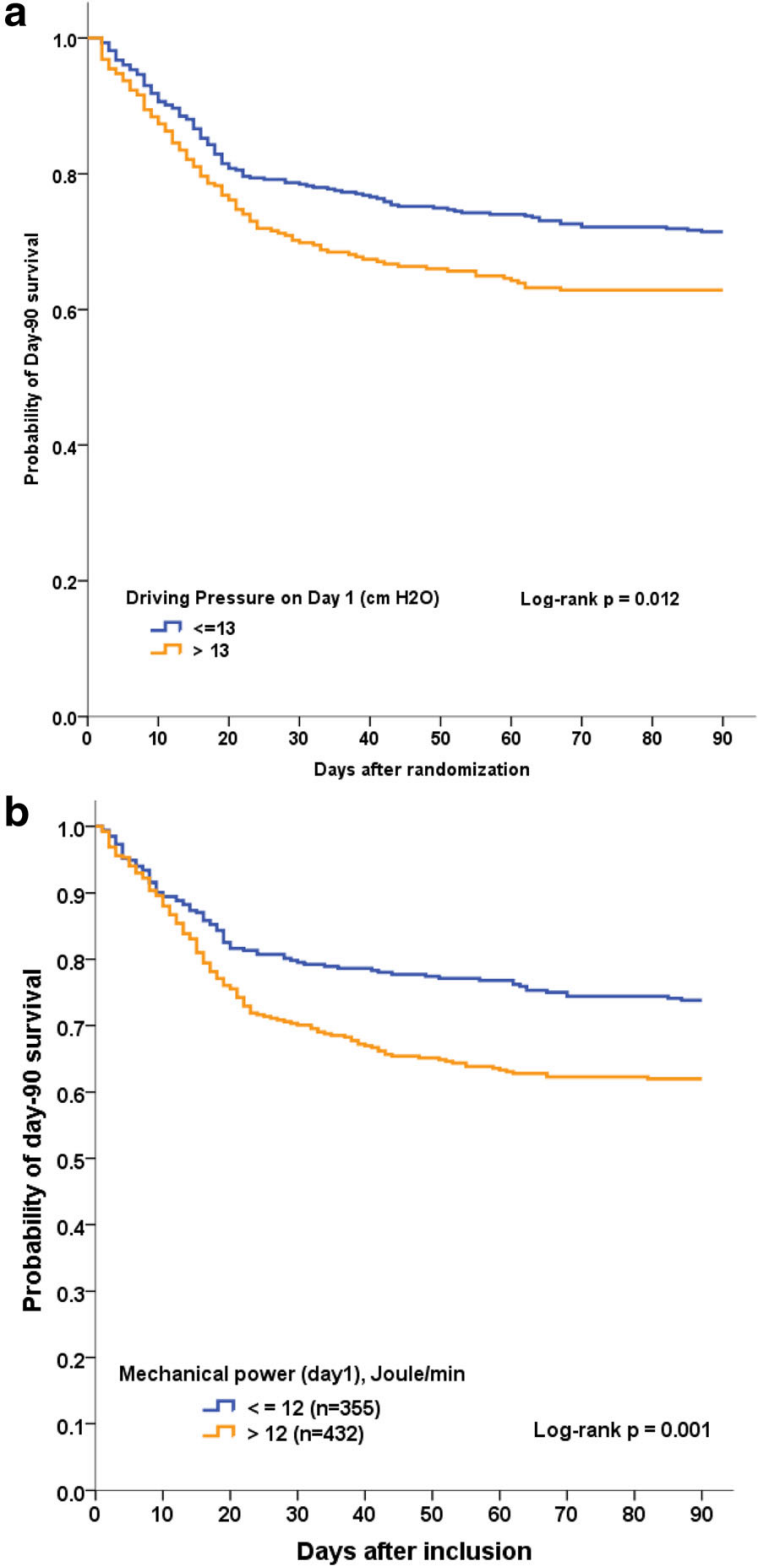
Kaplan-Meier graphs of the probability of survival for 90 days after inclusion in patients with acute respiratory distress syndrome, according to driving pressure (**a**) and mechanical power (**bb**) at day 1. The curves were compared using the log rank test

The Kaplan-Meier graphs describing the probability of survival from inclusion to 90 days for ΔPrs above or below 13 cm H_2_O and for mechanical power above or below 12 J/min at day 1 are shown in Fig. 2. The survival was significantly higher in patients with ΔPrs ≤13 cm H_2_O at day 1 than in those with ΔPrs >13 cm H_2_O and in patients with mechanical power ≤12 J/min at day 1 than in those with mechanical power >12 J/min. Survival was significantly higher in patients with Pplat,rs <23 cm H_2_O than in those with Pplat,rs ≥23 cm H_2_O and higher in patients with Crs <31 ml/cmH_2_O than in those with Crs ≥31 ml/cmH_2_O (see Additional file 5).

**Fig. 3:**
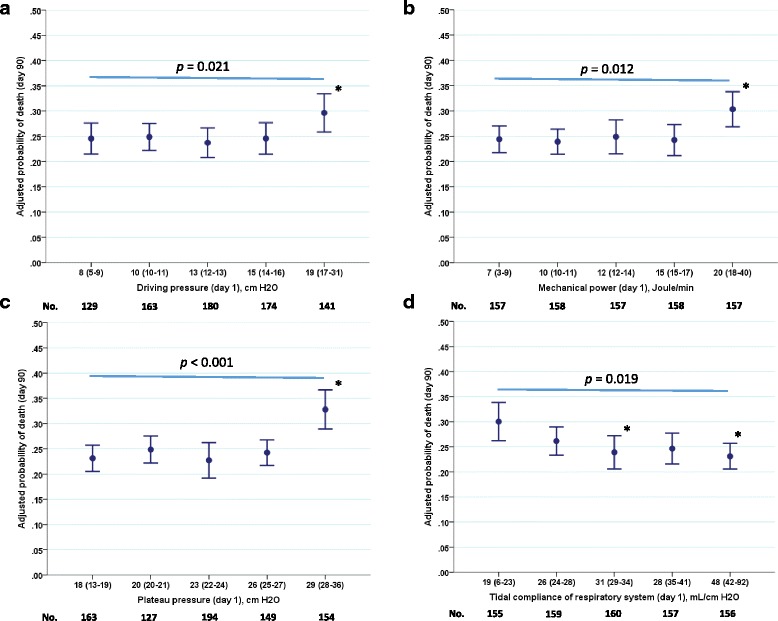
Unadjusted mortality at day 90 across quintiles of driving pressure (**a**), mechanical power (**b**), Pplat,rs (**c**) and Crs (**d**). The *bars* are standard error of the mean (SEM). The numbers below the *x axis* are the numbers of patients in each quintile. *P* < 0.001 across quintiles (analysis of variance). *P < 0.05 versus the first quintile

Whereas the unadjusted probabilities of survival were linearly related to quintiles of ΔPrs, mechanical power, Pplat,rs, and Crs (Fig. 1), their corresponding adjusted counterparts displayed a threshold in the vicinity of 15 cmH_2_O, 15 J/min, 26 cmH_2_O, and 26 ml/cmH_2_O, respectively (Fig. 3). The adjusted survival curves derived from the Cox regression analysis are shown for ΔPrs and mechanical power (see Additional file 6) and for Pplat,rs and Crs (see Additional file 7).

## Discussion

The main findings of the present study in our unique cohort were that: (1) ΔPrs was significantly associated with patient outcome after controlling for confounding factors, (2) survival was significantly higher in patients with ΔPrs ≤13 cm H_2_O at day 1, (3) survival was significantly higher in patients with mechanical power ≤12 J/min at day 1 and in patients with Pplat < 23 cmH_2_O, and (4) the information given by ΔPrs and Crs is similar. Our main finding was that with V_T_ and Pplat controlled, ΔPrs brings little more additional information independently on Pplat and Crs.

### Driving pressure

Rather than confirming the results of Amato et al. [3], the present findings disclosed the limitation of the prognostic role of ΔPrs when Plat,rs, V_T_, and PEEP are strictly controlled and set according to the ARMA trial. However, we found that the HR of ΔPrs was similar in Amato’s study and in the present investigation. In Amato’s study, in the Cox analysis ΔPrs was associated with 41% increased risk of mortality among the 3080 patients used in the combined analysis [3]. In this study, the authors used a 1-SD increment in ΔPrs for calculating HR, which represented 7 cmH_2_O. Thus, when calculating the HR for 1 cmH_2_O increment, this was associated with a HR of 1.049, which is very close to the present result, as shown in Table 2.

In our study, per each cm H_2_O increase, ΔPrs was associated with 5% increase in the risk of death, which is in the same order of magnitude as Pplat,rs, which was also significantly associated with mortality. PEEP and V_T_ were not significantly associated with mortality in the present cohort, whilst these were associated with a significant 2% and 3% increase in mortality per 1 cm H_2_O and per 1 ml/kg PBW, respectively, in Amato’s study [3]. This can be explained by the narrower range of PEEP and V_T_ used in our cohort. Therefore, in contrast to Amato’s study [3] our findings did not identify ΔPrs as the strongest predictor of death as compared to V_T_, Crs, and Pplat,rs. To explore this finding further, we used a model-building strategy that consisted of a series of Cox models, which included the collinear variables two-by-two (with their interaction) and these were then compared with the corresponding Cox models that used the collinear variable alone. This strategy showed that ΔPrs and Pplat,rs each provides different information related to patient outcome. However, interaction between them was present, statistically meaning that the effect of each of them on outcome was dependent on the level of the other. In other words, the effect of one covariate modifies the effect of the other on the outcome. When ΔPrs and mechanical power were analyzed two-by-two, ΔPrs remained significant but mechanical power did not. That means that ΔPrs conveys specific information. When ΔPrs and Crs were analyzed together neither of them remained statistically significantly associated with patient outcome. That means that the same information carried by Crs is also carried by ΔPrs. Both shared the same information. The same result, and hence, the same interpretation also applied for Pplat,rs and Crs.

The Lung Safe study [8] was a prospective international observational investigation in 50 countries, in which data were collected for over 2377 patients with ARDS in the winter season. In 703 of these patients data were available to analyze the rate of mortality at the time of hospital discharge over the range of ΔPrs and Pplat,rs. The mortality rate increased linearly with increasing ΔPrs with no threshold. The slope of the increase in mortality over ΔPrs quintiles was steeper than that pertaining to Pplat,rs in the Lung safe study, whereas the slopes were similar in the present study. However, V_T_ was not maintained at 6 ml/kg in these two studies [3, 8] which is at variance with the present study. Furthermore, in the Lung Safe study Pplat,rs was measured in only 40% of the patients [8], a fact that has been highlighted [9, 10].

ΔPrs ranged between 5 and 31 cm H_2_O in our cohort (Fig. 1), which is comparable to the range of 7–32 cm H_2_O in the Amato study, but wider than in the Lung Safe study (9–25 cm H_2_O). It should be stressed that in the Amato study [3] the effect of ΔPrs was related to the adjusted relative risk of death, whereas in our study, as in the Lung Safe study, the probability of death was analyzed. Moreover only patients with a P/F ratio <150 mmHg were included. ΔPrs was also reported to be associated with death in a recent large multicenter cohort of patients with ARDS who had acute cor pulmonale [11].

A more relevant analysis of the data on ΔPrs would require the knowledge of the transpulmonary ΔP (ΔP_L_). Talmor et al. found that the reduction in ΔP_L_ was higher in an esophageal pressure-guided group than in a control group, and that ΔP_L_ reduction was higher in survivors than in nonsurvivors, whereas ΔPrs was similar in both experimental and control groups and in survivors and nonsurvivors [12], confirming that the compliance of the chest wall is a key parameter in interpreting ΔPrs and its components. The role of ΔP_L_ to optimize the use of mechanical ventilation in the prone position should be further investigated, in particular regarding PEEP selection [13], by using a physiological approach [14].

### Mechanical power

The concept that the magnitude of energy transferred from the ventilator into the lung may contribute to VILI has recently arisen and has been confirmed in an experimental study in normal pigs receiving a combination of a large number of V_T_ and respiratory rates [15]. In this study mechanical power of 12 J/min was found to promote VILI. In the present study, our secondary goal was to explore whether the mechanical power was associated with the outcome. We found that this was the case and the threshold of 12 J/min was associated with significant distinct probabilities of survival. Interestingly, the median value of mechanical power in the present cohort was the same as that found experimentally as the threshold above which VILI occurred [15]. We also found that the value of the mechanical power in J/min was very close to that of ΔPrs in cm H_2_O. The relevance of the present data on mechanical power should be confirmed by further investigations. Should mechanical power be confirmed as a significant independent predictor of survival its computation at the bedside should be recommended. Recently, Gattinoni et al. [16] proposed using the first-order equation to compute mechanical power. Our present approach is much simpler and can be easily implemented at the bedside.

The probability of survival in our study was expressed as unadjusted and adjusted, taking into account the covariates selected by the Cox models. This explains the difference between the data shown in Fig. 1 and Fig. 3. In the former, a linear relationship was observed between survival and ΔPrs, mechanical power, Pplat,rs and Crs. This suggests there is no safe dose of mechanical ventilation. However, when the survival was adjusted with covariates, a threshold was disclosed for the survival across quintiles.

### Limitations and strengths

Our study was limited by: (1) the fact that data were collected from two positive trials where survival was markedly affected by the experimental approach subjected to randomization; (2) as in other trials in patients with ARDS, more than 60% of patients meeting the criteria for ARDS were excluded from enrollment into the trials; and (3) lack of generalizability, as patients with PaO2/FiO2 > 150 mmHg at 24 hours were excluded from the analysis. However, as discussed previously, our ARDS sample was more homogeneous in terms of the ventilator settings used and the present results were highly significant.

### Clinical implications

The main clinical message from our data is that if V_T_ and Pplat,rs are strictly maintained to 6 ml/kg predicted body weight and below 28–30 cmH_2_O, ΔPrs shares the same information as Pplat,rs about the association with day 90-mortality. Management of patients with ARDS is an ongoing process that combines physiologic [14] and pragmatic information [17]. The use of ΔPrs to manage patients as a therapeutic target should be part of the research agenda in ARDS [18]. However, better knowledge of the physiologic meaning of ΔPrs is mandatory, to make sure that ΔPrs is a relevant tool to set the ventilator adequately in ARDS patients, as it has been done for PEEP selection [19–23]. As an example, it has been shown that ΔPrs correlates with lung stress and, hence could detect over-distension [24].

## Conclusions

When lung protective mechanical ventilation is applied to patients with ARDS, ΔPrs, Crs, and Pplat were risk factors for mortality.

## Additional files

Additional file 1: Table S1. Multivariate Cox regression analysis for factors on day 1 including mechanical power associated with ARDS mortality at day 90. (DOC 33 kb)
Additional file 2: Table S2. Multivariate Cox regression analysis for factors on day 1 including plateau pressure associated with ARDS mortality at day 90. (DOC 33 kb)
Additional file 3: Table S3. Multivariate Cox regression analysis for factors on day 1 including compliance of the respiratory system associated with ARDS mortality at day 90. (DOC 33 kb)
Additional file 4: Table S4. Multivariate Cox regression analysis for factors on day 1 including a couple of collinear variables associated with ARDS mortality at day 90. (DOCX 19 kb)
Additional file 5: Figure S1. Kaplan-Meier graphs of the probability of survival over 90 days after inclusion of patients with ARDS according to Pplat,rs and Crs. The curves were compared by using the log rank test. (PPTX 92 kb)
Additional file 6: Figure S2. Adjusted probability of survival derived from the Cox model according to ΔPrs (**A**) and mechanical power (**B**). (PPTX 89 kb)
Additional file 7: Figure S3. Adjusted probability of survival derived from the Cox model according Pplat,rs (**A**) and Crs (**B**). (PPTX 90 kb)

## Abbreviations

Crs: compliance of the respiratory system
FIO2: oxygen fraction in air
HR: hazard ratio
NMBA: neuromuscular blocking agent
PaCO2: artial pressure of carbon dioxide in arterial blood
PaO2: partial pressure of oxygen in arterial blood
PEEP: positive end-expiratory pressure
PEEPtot,rs: total peep of the respiratory system
pH: potential of hydrogen
Pplat,rs: plateau pressure of the respiratory system
SAPS: simplified acute physiology score
SD: standard deviation
SOFA: sequential organ failure assessment
VILI: ventilator-induced lung injury
VT: tidal volume
ΔPL: transpulmonary driving pressure
ΔPrs: driving pressure of the respiratory system
