# Supplementary material for: Effect of driving pressure on mortality in ARDS patients during lung protective mechanical ventilation in two randomized controlled trials
Source: Crit Care. 2016 Nov 29;20:384. doi: 10.1186/s13054-016-1556-2 (PMC5126997; doi:10.1186/s13054-016-1556-2)
Supplement: Additional file 1: Table S1. — Multivariate Cox regression analysis for factors on day 1 including mechanical power associated with ARDS mortality at day 90. (DOC 33 kb) [file 13054_2016_1556_MOESM1_ESM.doc]

Additional file 1. Table S1. Multivariate Cox regression analysis for factors on day 1 including mechanical power associated with ARDS mortality at day 90

| **Variables** | | **Hazard Ratio (95% CI)** | *p* |
| --- | --- | --- | --- |
| Age, per year | 1.04 (1.03 – 1.05) | | <0.001 |
| SOFA score on day 1, per unit | 1.06 (1.02 – 1.10) | | <0.001 |
| Continuous NMBA as allocation group, (reference is yes) | 0.67 (0.47 – 0.94) | | 0.022 |
| Prone position as allocation group (reference is yes) | 0.66 (0.46 – 0.94) | | 0.025 |
| PaO_2_/FiO_2_ on day 1, per unit | 1.00 (0.99 – 1.01) | | 0.841 |
| Arterial pH on day 1, per unit | 0.052 (0.008 – 0.334) | | 0.002 |
| Lactate on day 1, per unit | 14.53 (1.12 – 188.20) | | 0.041 |
| Interaction lactate * arterial pH on day 1, per unit | 0.69 (0.49 – 0.99) | | 0.044 |
| Mechanical power on day 1, per unit | 1.03 (1.01 – 1.05) | | 0.023 |

CI, confidence intervals; SOFA, Sequential Organ Failure Assessment; NMBA, neuromuscular blocking agent. Day 1 was defined as the 24 hours following the inclusion. Mechanical power was calculated as the product of driving pressure, tidal volume and respiratory rate.
