# Supplementary material for: Effect of driving pressure on mortality in ARDS patients during lung protective mechanical ventilation in two randomized controlled trials
Source: Crit Care. 2016 Nov 29;20:384. doi: 10.1186/s13054-016-1556-2 (PMC5126997; doi:10.1186/s13054-016-1556-2)
Supplement: Additional file 2: Table S2. — Multivariate Cox regression analysis for factors on day 1 including plateau pressure associated with ARDS mortality at day 90. (DOC 33 kb) [file 13054_2016_1556_MOESM2_ESM.doc]

Additional file 2. Table S2. Multivariate Cox regression analysis for factors on day 1 including plateau pressure associated with ARDS mortality at day 90

| **Variables** | **Hazard Ratio (95% CI)** | ***p*** |
| --- | --- | --- |
| Age, per year | 1.04 (1.03 – 1.05) | <0.001 |
| SOFA score on day 1, per unit | 1.07 (1.03– 1.11) | <0.001 |
| Continuous NMBA as allocation group, (reference is yes) | 0.65 (0.46 – 0.92) | 0.016 |
| Prone position as allocation group, (reference is yes) | 0.67 (0.46 – 0.96) | 0.029 |
| Respiratory rate on day 1, per unit | 1.01 (0.98 – 1.03) | 0.670 |
| PaO_2_/FiO_2_ on day 1, per unit | 1.00 (0.99 – 1.01) | 0.754 |
| Arterial pH on day 1, per unit | 0.08 (0.012 – 0.534) | 0.009 |
| Lactate on day 1, per unit | 21.08 (1.48 – 299.51) | 0.024 |
| Interaction lactate * arterial pH on day 1, per unit | 0.66 (0.46– 0.95) | 0.027 |
| Plateau pressure on day 1, per unit | 1.04 (1.01 – 1.08) | 0.008 |

CI, confidence intervals; SOFA, Sequential Organ Failure Assessment; NMBA, neuromuscular blocking agents. Day 1 was defined as the 24 hours following the inclusion
