# Supplementary material for: Effect of driving pressure on mortality in ARDS patients during lung protective mechanical ventilation in two randomized controlled trials
Source: Crit Care. 2016 Nov 29;20:384. doi: 10.1186/s13054-016-1556-2 (PMC5126997; doi:10.1186/s13054-016-1556-2)
Supplement: Additional file 3: Table S3. — Multivariate Cox regression analysis for factors on day 1 including compliance of the respiratory system associated with ARDS mortality at day 90. (DOC 33 kb) [file 13054_2016_1556_MOESM3_ESM.doc]

**Additional file 3.** Table S3. Multivariate Cox regression analysis for factors on day 1 including compliance of the respiratory system associated with ARDS mortality at day 90

| **Variables** | **Hazard Ratio (95% CI)** | ***p*** |
| --- | --- | --- |
| Age, per year | 1.04 (1.03 – 1.05) | <0.001 |
| SOFA score on day 1, per unit | 1.07 (1.02 – 1.11) | 0.001 |
| Continuous NMBA as allocation group, (reference is yes) | 0.66 (0.47 – 0.94) | 0.022 |
| Prone position as allocation group, (reference is yes) | 0.57 (0.38 – 0.85) | 0.006 |
| Respiratory rate on day 1, per unit | 1.01 (0.98 – 1.04) | 0.612 |
| PaO_2_/FiO_2_ on day 1, per unit | 1.00 (0.99 – 1.01) | 0.838 |
| Arterial pH on day 1, per unit | 0.064 (0.009 – 0.452) | 0.006 |
| Lactate on day 1, per unit | 21.89 (1.37 – 351002) | 0.029 |
| Interaction lactate * arterial pH on day 1, per unit | 0.66 (0.45– 0.97) | 0.033 |
| Tidal compliance on day 1, per unit | 0.985 (0.972 – 0.999) | 0.029 |

CI, confidence intervals; SOFA, Sequential Organ Failure Assessment; NMBA, neuromuscular blocking agents. Day 1 was defined as the 24 hours following the inclusion. Tidal compliance of respiratory system was calculated as the ratio of tidal volume to driving pressure.
