# Supplementary material for: Effect of driving pressure on mortality in ARDS patients during lung protective mechanical ventilation in two randomized controlled trials
Source: Crit Care. 2016 Nov 29;20:384. doi: 10.1186/s13054-016-1556-2 (PMC5126997; doi:10.1186/s13054-016-1556-2)
Supplement: Additional file 4: Table S4. — Multivariate Cox regression analysis for factors on day 1 including a couple of collinear variables associated with ARDS mortality at day 90. (DOCX 19 kb) [file 13054_2016_1556_MOESM4_ESM.docx]

**Additional file 4.** Table S4. Multivariate Cox regression analysis for factors on day 1 including a couple of collinear variables and associated with ARDS mortality at day 90

| **Model 1. Couple is Driving pressure and Plateau pressure on day 1** | | | |
| --- | --- | --- | --- |
| **Variables** | **Hazard Ratio (95% CI)** | ***p*** | |
| Age, per year | 1.04 (1.03 – 1.05) | <0.001 |  |
| SOFA score on day 1, per unit | 1.07 (1.03 – 1.11) | <0.001 |  |
| Continuous NMBA as allocation group, (reference is yes) | 0.64 (0.45 – 0.90) | 0.011 |  |
| Prone position as allocation group, (reference is yes) | 0.68 (0.47 – 0.98) | 0.038 |  |
| Respiratory rate on day 1, per unit | 1.01 (0.98 – 1.03) | 0.656 |  |
| Driving pressure on day 1, per unit | 1.31 (1.07 – 1.61) | 0.010 |  |
| Plateau pressure on day 1, per unit | 1.13 (1.02 – 1.26) | 0.021 |  |
| Interaction Driving pressure * Plateau pressure on day 1, per unit | 0.99 (0.98 – 0.99) | 0.019 |  |
| PaO_2_/FiO_2_ on day 1, per unit | 1.00 (0.99 – 1.02) | 0.991 |  |
| Arterial pH on day 1, per unit | 0.045 (0.006 – 0.329) | 0.002 |  |
| Lactate on day 1, per unit | 17.75 (1.21 – 259.96) | 0.036 |  |
| Interaction lactate * arterial pH on day 1, per unit | 0.67 (0.46 – 0.98) | 0.039 |  |
| **Model 2. Couple is Mechanical power and Driving pressure on day 1** | | |  |
| **Variables** | **Hazard Ratio (95% CI)** | ***p*** |  |
| Age, per year | 1.04 (1.03 – 1.05) | <0.001 |  |
| SOFA score on day 1, per unit | 1.07 (1.03 – 1.11) | 0.001 |  |
| Continuous NMBA as allocation group, (reference is yes) | 0.63 (0.45 – 0.90) | 0.011 |  |
| Prone position as allocation group, (reference is yes) | 0.68 (0.47 – 0.97) | 0.036 |  |
| Driving pressure on day 1, per unit | 1.12 (1.03 – 1.22) | 0.011 |  |
| Mechanical power on day 1, per unit | 1.09 (0.99 – 1.19) | 0.070 |  |
| Interaction Driving pressure * Mechanical power on day 1, per unit | 0.995 (0.990 – 1.000) | 0.062 |  |
| PaO_2_/FiO_2_ on day 1, per unit | 1.00 (0.99 – 1.01) | 0.980 |  |
| Arterial pH on day 1, per unit | 0.042 (0.006 – 0.281) | 0.001 |  |
| Lactate on day 1, per unit | 18.82 (1.21 – 292.62) | 0.036 |  |
| Interaction lactate * arterial pH on day 1, per unit | 0.67 (0.46 – 0.98) | 0.039 |  |

**Table 5 ESM (continued).**

| **Model 3. Couple is Driving pressure and Tidal compliance of respiratory system on day 1** | | |
| --- | --- | --- |
| **Variables** | **Hazard Ratio (95% CI)** | ***p*** |
| Age, per year | 1.04 (1.03 – 1.05) | <0.001 |
| SOFA score on day 1, per unit | 1.07 (1.03 – 1.11) | <0.001 |
| Continuous NMBA as allocation group, (reference is yes) | 0.64 (0.45 – 0.91) | 0.012 |
| Prone position as allocation group, (reference is yes) | 0.68 (0.47 – 0.97) | 0.036 |
| Respiratory rate on day 1, per unit | 1.00 (0.98 – 1.03) | 0.696 |
| Driving pressure on day 1, per unit | 0.99 (0.92 – 1.08) | 0.932 |
| Crs on day 1, per unit | 0.98 (0.94 – 1.01) | 0.192 |
| Interaction Driving pressure * Crs on day 1, per unit | 1.002 (0.999-1.005) | 0.268 |
| PaO_2_/FiO_2_ on day 1, per unit | 1.00 (0.99 – 1.01) | 0.811 |
| Arterial pH on day 1, per unit | 0.050 (0.008 – 0.335) | 0.002 |
| Lactate on day 1, per unit | 16.66 (1.20 – 231.84) | 0.036 |
| Interaction lactate * arterial pH on day 1, per unit | 0.68 (0.47 – 0.98) | 0.040 |
| **Model 4. Couple is Plateau pressure and Tidal compliance of respiratory system on day 1** | | |
| **Variables** | **Hazard Ratio (95% CI)** | ***p*** |
| Age, per year | 1.04 (1.03 – 1.05) | <0.001 |
| SOFA score on day 1, per unit | 1.07 (1.03 – 1.11) | <0.001 |
| Continuous NMBA as allocation group, (reference is yes) | 0.64 (0.45 – 0.91) | 0.013 |
| Prone position as allocation group, (reference is yes) | 0.66 (0.46 – 0.95) | 0.026 |
| Respiratory rate on day 1, per unit | 1.00 (0.98 – 1.03) | 0.812 |
| Plateau pressure on day 1, per unit | 0.97 (0.89 – 1.06) | 0.535 |
| Crs on day 1, per unit | 0.95 (0.89 – 1.01) | 0.105 |
| Interaction Plateau pressure * Crs on day 1, per unit | 1.002 (0.999 – 1.005) | 0.169 |
| PaO_2_/FiO_2_ on day 1, per unit | 1.00 (0.99 – 1.01) | 0.861 |
| Arterial pH on day 1, per unit | 0.065 (0.009 – 0.449) | 0.006 |
| Lactate on day 1, per unit | 21.01 (1.43 – 307.76) | 0.026 |
| Interaction lactate * arterial pH on day 1, per unit | 0.66 (0.46 – 0.96) | 0.029 |

CI, confidence intervals; SOFA, Sequential Organ Failure Assessment; NMBA, neuromuscular blocking agent, Crs: tidal compliance of respiratory system. Day 1 was defined as the 24 hours following the inclusion. Driving pressure was calculated as the difference between Plateau pressure and applied PEEP. Mechanical power was calculated as the product of driving pressure, tidal volume and respiratory rate. Tidal compliance of respiratory system was calculated as the ratio of tidal volume to driving pressure.
