# Supplementary figures and images for: Effect of driving pressure on mortality in ARDS patients during lung protective mechanical ventilation in two randomized controlled trials
Source: Crit Care. 2016 Nov 29;20:384. doi: 10.1186/s13054-016-1556-2 (PMC5126997; doi:10.1186/s13054-016-1556-2)

## Slide 1
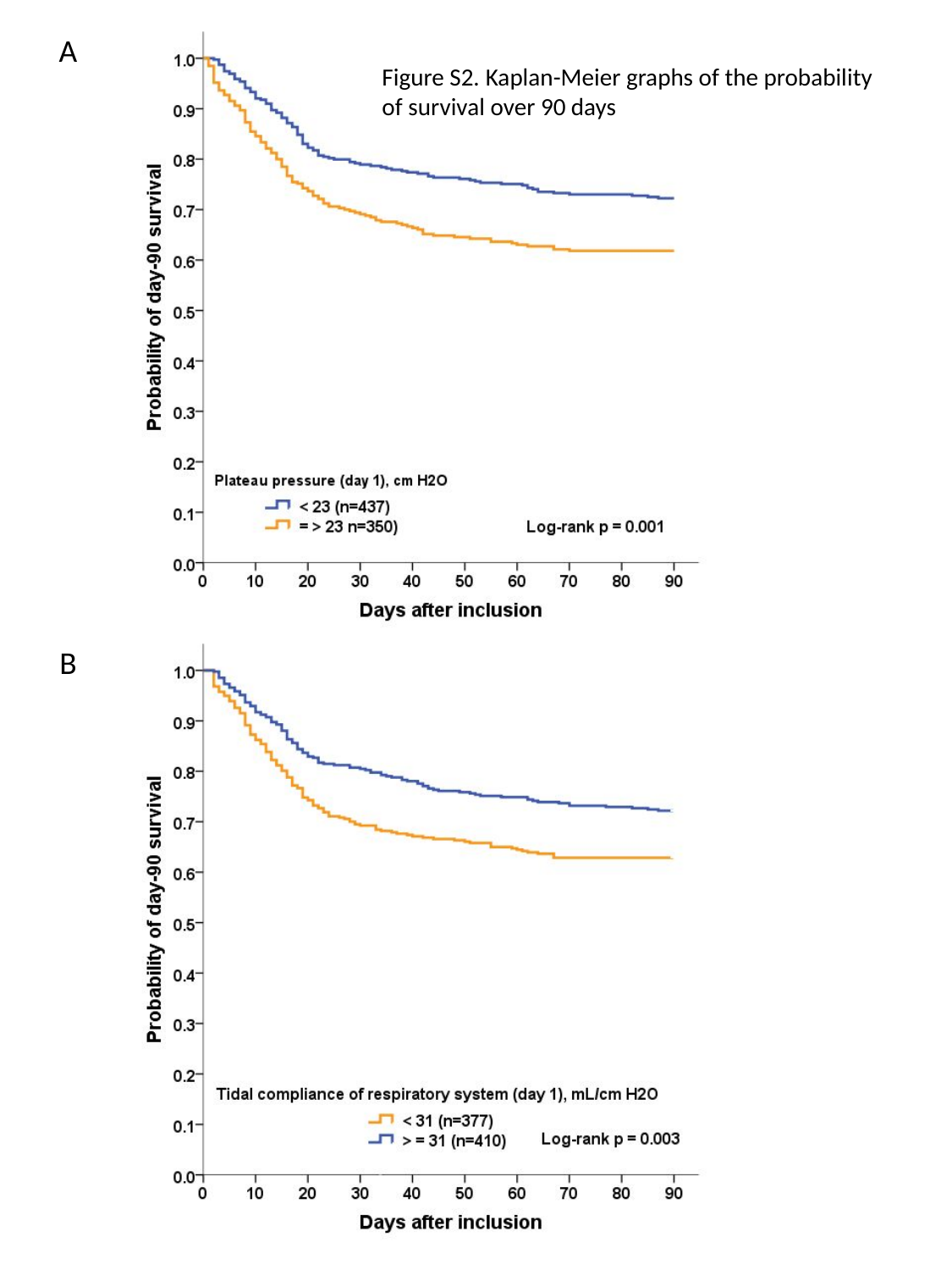

A
Figure S2. Kaplan-Meier graphs of the probability
of survival over 90 days
B

Supplement: Additional file 5: Figure S1. — Kaplan-Meier graphs of the probability of survival over 90 days after inclusion of patients with ARDS according to Pplat,rs and Crs. The curves were compared by using the log rank test. (PPTX 92 kb) [file 13054_2016_1556_MOESM5_ESM.pptx]

## Slide 1
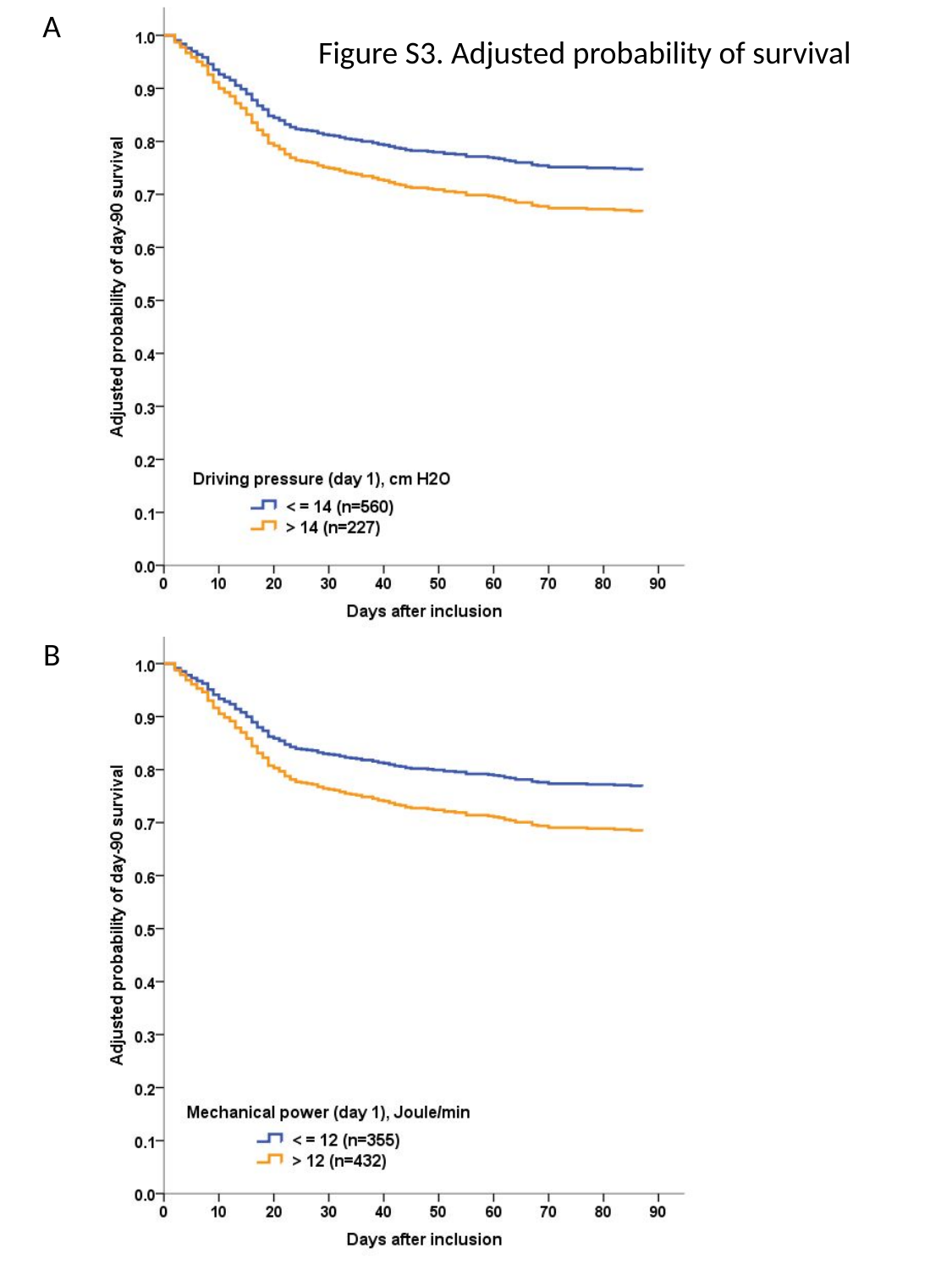

A
Figure S3. Adjusted probability of survival
B

Supplement: Additional file 6: Figure S2. — Adjusted probability of survival derived from the Cox model according to ΔPrs (A) and mechanical power (B). (PPTX 89 kb) [file 13054_2016_1556_MOESM6_ESM.pptx]

## Slide 1
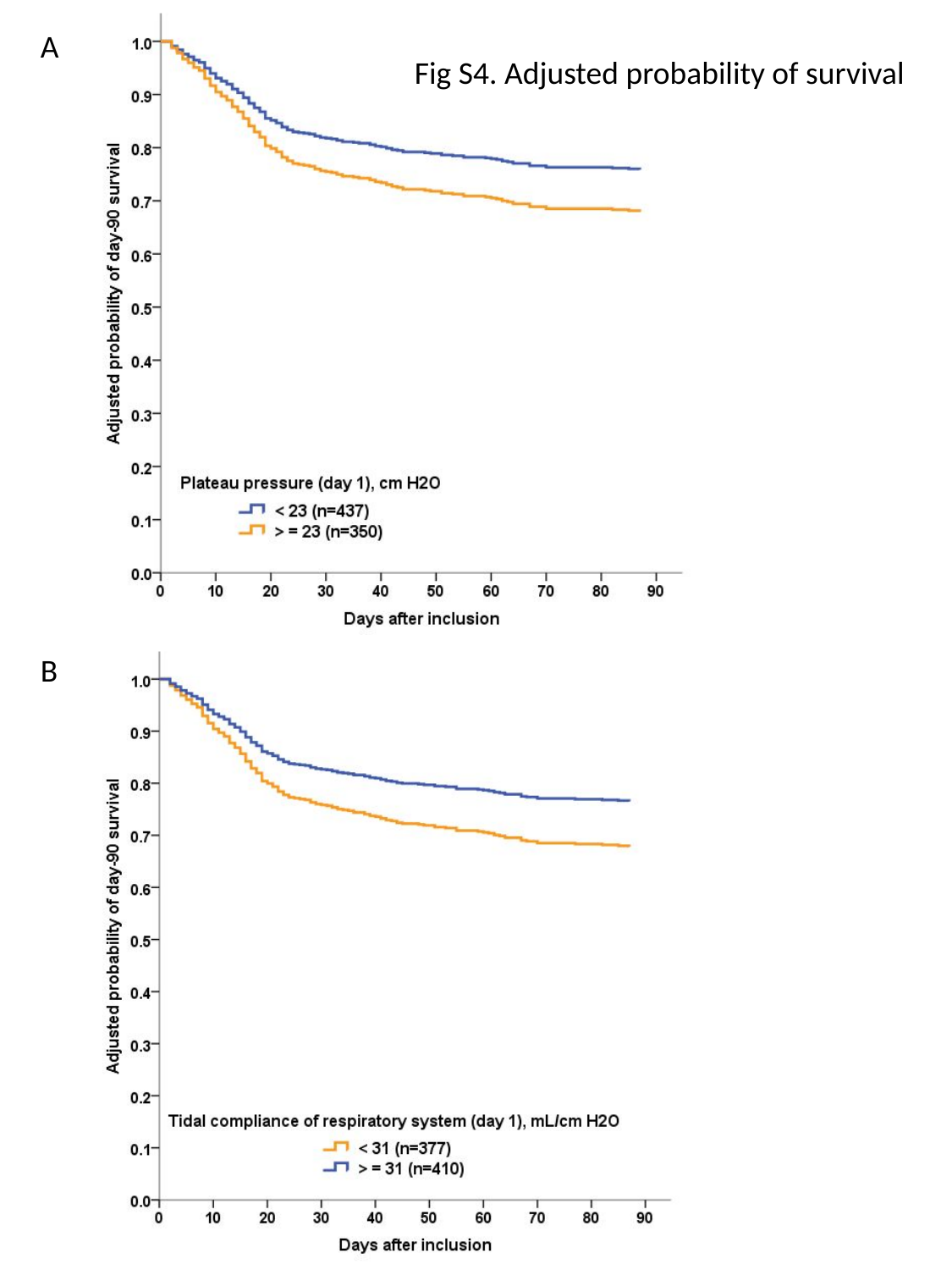

A
Fig S4. Adjusted probability of survival
B

Supplement: Additional file 7: Figure S3. — Adjusted probability of survival derived from the Cox model according Pplat,rs (A) and Crs (B). (PPTX 90 kb) [file 13054_2016_1556_MOESM7_ESM.pptx]
